# Bexar County Medical Society

**Published:** 1903-09

**Authors:** 


### Society Notes.

### Bexar County Medical Society.

The newly organized Bexar County Medical Society held its first regular meeting and smoker in San Antonio on Thursday night, August 6th. Vice-President W. M. Wolf presided, and sixty-five members were present. Brigadier-General Peter J. A. Cleary, who, since coming to San Antonio, has attended every regular meeting of the local society, and who has at all times been an enthusiastic member, was elected to honorary life membership. Dr. Ferdinand Herff was also honored in a similar manner. The nominations of both General Cleary and Dr. Herff were greeted with applause.

The following resolutions of respect to the memory or Dr. B. E. Hadra were adopted:

"Whereas, In the mysterious wisdom of Divine Providence Dr. B. E. Hadra has answered the last call; be it

"*Resolved* by the Bexar County Medical Society, That in the death of Dr. Hadra the medical profession of the world has lost one of its bright lights, one of its strongest and ablest supporters; that the suffering poor have lost a noble and true friend, who was ever ready to answer their call of distress; and that his associates have lost a companion whose brilliant genius, quick wit, and genial good fellowship won the admiration and love of all who knew him.

"*Resolved further,* That this society tenders its warm sympathy to the bereaved widow and children, and that a copy of these resolutions be sent to the family and to the press, and be spread upon the minutes of this society.

"J. S. Lankford,
"P. J. A. Cleary,
"C. E. R. King,
"F. Paschal,
"Adolph Herff."

Dr. G. Graham Watts reported an unusual case of abscess of the liver, illustrating the great difficulty of diagnosis sometimes experienced in this surgical disease. Discussion by Drs. F. E. Young, Adolph Herff, Frank Paschal, B. F. Kingsley, R. A. Goeth, General Cleary and Major C. F. Mason, U. S. A.

Dr. James Hall Bell delivered a timely and interesting address on "The American Medical Association Reorganization Plan," in which he outlined the history of the movement and emphasized its salient features.

In further discussing the matter Dr. W. B. Russ emphasized the fact that its object is not alone to promote the advancement of scientific medicine, but also to serve both the profession and the public by putting a stop to the occurrence of the wretched and senseless quarrels and misunderstandings between medical men, the tendency of which is to greatly impair the profession's usefulness to the public and to degrade and pauperize its members individually and collectively.

Secretary T. P. Lloyd and Treasurer T. E. Moore reported the finances of the society to be in first-class condition, and that almost every reputable physician in the county has applied for membership.

The officers of the society are: T. T. Jackson, President; W. M. Wolf, Vice-President; T. P. Lloyd, Secretary; T. E. Moore, Treasurer; J. S. Lankford, D. Berrey and W. E. Luter, Censors.

---

